# 1-[(*E*)-Anthracen-9-yl­methyl­idene]-2-(2,4-di­nitro­phen­yl)hydrazine

**DOI:** 10.1107/S1600536813009197

**Published:** 2013-04-13

**Authors:** Joana de A. e Silva, Consuelo Yuste-Vivas, Abilio J. F. N. Sobral, Manuela Ramos Silva

**Affiliations:** a Chemistry Department, University of Coimbra, P-3004-530 Coimbra, Portugal; b CEMDRX, Physics Department, University of Coimbra, P-3004-516 Coimbra, Portugal

## Abstract

In the title Schiff base, C_21_H_14_N_4_O_4_, the dihedral angle between the two nitro groups and the central benzene ring are 83.6 (5) and 2.6 (6)°. The anthracene ring system and the benzene ring make a dihedral angle of 0.7 (2)°. Intra­molecular N—H⋯O and C—H⋯N hydrogen bonds occur. In the crystal, C—H⋯O hydrogen bonds link the mol­ecules, forming chains along the *b*-axis direction.

## Related literature

For general background to hydrazone derivatives, see: Kahwa *et al.* (1986 ▶). For the structures of 2,4-di­nitro­phenyl­hydrazine and 9-anthraldehyde, see: Okabe *et al.* (1993 ▶) and Trotter (1959 ▶), respectively. 
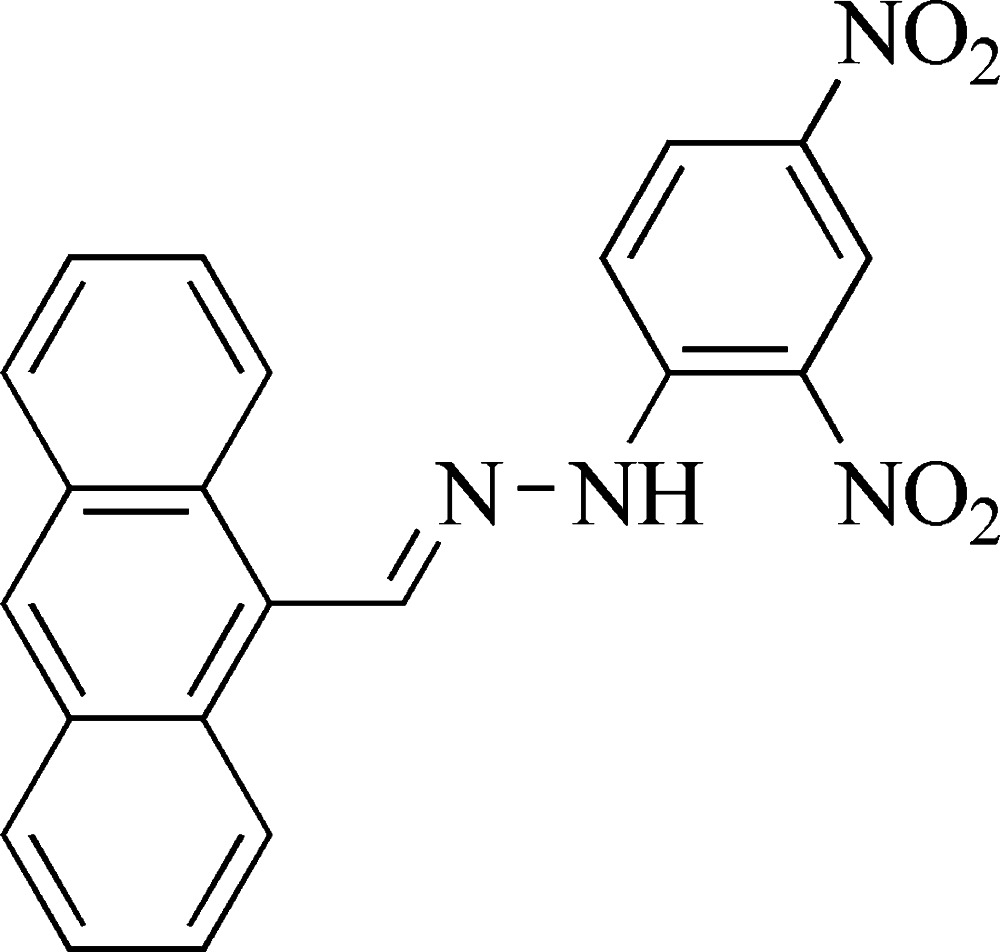


## Experimental

### 

#### Crystal data

C_21_H_14_N_4_O_4_
*M* *_r_* = 386.36
Orthorhombic, 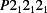

*a* = 5.6355 (4) Å
*b* = 8.1597 (5) Å
*c* = 36.794 (2) Å
*V* = 1691.95 (19) Å^3^
*Z* = 4
Mo *K*α radiation
μ = 0.11 mm^−1^
*T* = 293 K
0.08 × 0.02 × 0.01 mm

#### Data collection

Bruker APEXII CCD area-detector diffractometer
Absorption correction: multi-scan (*SADABS*; Sheldrick, 2000 ▶) *T*_min_ = 0.764, *T*_max_ = 0.999
18174 measured reflections
3708 independent reflections
1466 reflections with *I* > 2σ(*I*)
*R*_int_ = 0.132

#### Refinement

*R*[*F* ^2^ > 2σ(*F* ^2^)] = 0.074
*wR*(*F* ^2^) = 0.242
*S* = 0.88
3708 reflections
263 parameters
H-atom parameters constrained
Δρ_max_ = 0.25 e Å^−3^
Δρ_min_ = −0.22 e Å^−3^

### 

Data collection: *APEX2* (Bruker, 2003 ▶); cell refinement: *SAINT* (Bruker, 2003 ▶); data reduction: *SAINT*; program(s) used to solve structure: *SHELXS97* (Sheldrick, 2008 ▶); program(s) used to refine structure: *SHELXL97* (Sheldrick, 2008 ▶); molecular graphics: *DIAMOND* (Brandenburg, 2006 ▶); software used to prepare material for publication: *WinGX* publication routines (Farrugia, 2012 ▶).

## Supplementary Material

Crystal structure: contains datablock(s) I, global. DOI: 10.1107/S1600536813009197/bt6891sup1.cif

Structure factors: contains datablock(s) I. DOI: 10.1107/S1600536813009197/bt6891Isup2.hkl

Supplementary material file. DOI: 10.1107/S1600536813009197/bt6891Isup3.cml

Additional supplementary materials:  crystallographic information; 3D view; checkCIF report

## Figures and Tables

**Table 1:** Hydrogen-bond geometry (Å, °)

*D*—H⋯*A*	*D*—H	H⋯*A*	*D*⋯*A*	*D*—H⋯*A*
N3—H03⋯O4	0.86	1.99	2.617 (7)	129
C11—H11⋯O4^i^	0.93	2.47	3.251 (8)	142
C20—H20⋯N4	0.93	2.25	2.894 (8)	126

Symmetry code: (i) 
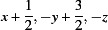
.

**Fig. 1.**
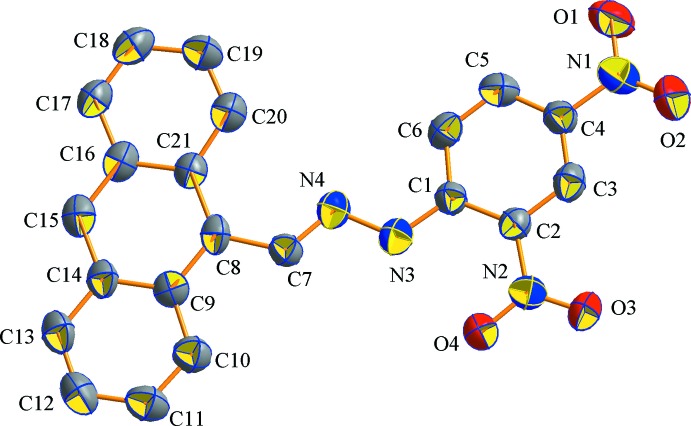
: Asymmetric unit of the title compound, showing the atom-labelling scheme and displacement ellipsoids drawn at the 50% probability level.

**Fig. 2.**
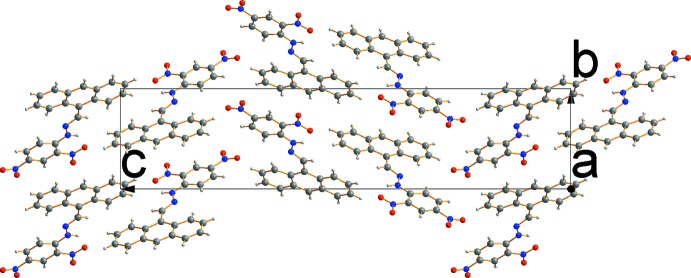
: View of the crystal packing of the title compound, projected along c.

**Fig. 3.**
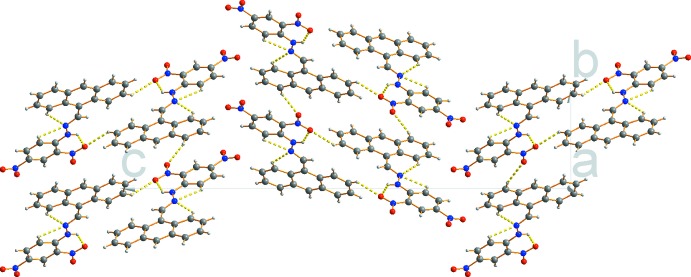
: A view showing part of the three-dimensional supramolecular network linked by weak hydrogen-bond interactions (yellow dotted lines).

## supplementary crystallographic information

### Comment

The title compound was synthesized as part of an investigation of the coordination properties of Schiff bases functioning as ligands. Metal complexes based on Schiff bases have been developed in biology and macromolecular chemistry in the last years (Kahwa *et al.*, 1986).

The three dimensional arrangement of the molecules is held together by weak hydrogen bonds interactions between C—H and nitro-oxygen atoms.

Each unit is almost planar with a maximum deviation of 0.179 (6) A for O2, bond lengths varying in the ranges of [1.331 (9)–1.463 (8), 1.215 (7)–1.242 (6), 1.294 (7)–1.461 (8) and 1.389 (7) A for C—C, N—O, C—N and N—N respectively] and bond angles agreeing with those for the initial ligands. Molecules grow along the *a*-axis giving layers in the plane *bc* with an ABAB disposition, as well as each A and B layers are actually an alternating double layer. Two neighbor units of compound 1 create an angle of 68.92 (3)° between them along the *c*-axis.

The angle between the two nitro groups and the central benzene ring by 83.6 (5) and 2.6 (6)°, and the angle between these two nitro groups is 11.1 (7)°. Dihedral angle between the two aromatic parts of the molecule are 179.7 (6) and -171.7 (6)°, for C8—C7—N4—N3 and C7—N4—N3—C1 respectively.

### Experimental

All reagents were obtained from commercial sources and used wirh no further purifications.

The compound was obtained when 1 g of (2,4-dinitrophenyl) hydrazine was dissolved in 5 mL of concentrated H_2_SO_4_.7.5 mL of water where added very slowly to the solution, after this were also added 25 mL of ethanol. In other flask, 4 mL of ethanol 0.05 g of anthracene-9-carbaldehyde where dissolved, and then, 1.80 mL of (2,4-dinitrophenyl)hydrazine was added to the solution. The two solutions were mixed and left to stand, at room temperature, for 24 h and then the solid compound was filtered., 049 g (52,7%)of the final product were obtained.

### Refinement

All H atoms could be located in a difference Fourier synthesis but were placed in calculated positions and refined as riding on their parent atoms, using *SHELXL97* (Sheldrick, 2008) defaults. Due to the absence of anomalous scatterers, the absolute structure could not be determined.

### Crystal data

**Table:**

C_21_H_14_N_4_O_4_	*F*(000) = 146
*M**_r_* = 386.36	*D*_x_ = 1.517 Mg m^−^^3^
Orthorhombic, *P*2_1_2_1_2_1_	Mo *K*α radiation, λ = 0.71073 Å
Hall symbol: P 2ac 2ab	Cell parameters from 1164 reflections
*a* = 5.6355 (4) Å	θ = 3.0–18.8°
*b* = 8.1597 (5) Å	µ = 0.11 mm^−^^1^
*c* = 36.794 (2) Å	*T* = 293 K
*V* = 1691.95 (19) Å^3^	Needle, colorless
*Z* = 4	0.08 × 0.02 × 0.01 mm

### Data collection

**Table:**

Bruker APEXII CCD area-detector diffractometer	3708 independent reflections
Radiation source: fine-focus sealed tube	1466 reflections with *I* > 2σ(*I*)
Graphite monochromator	*R*_int_ = 0.132
φ and ω scans	θ_max_ = 27.3°, θ_min_ = 3.3°
Absorption correction: multi-scan (*SADABS*; Sheldrick, 2000)	*h* = −7→7
*T*_min_ = 0.764, *T*_max_ = 0.999	*k* = −10→9
18174 measured reflections	*l* = −46→46

### Refinement

**Table:**

Refinement on *F*^2^	Secondary atom site location: difference Fourier map
Least-squares matrix: full	Hydrogen site location: inferred from neighbouring sites
*R*[*F*^2^ > 2σ(*F*^2^)] = 0.074	H-atom parameters constrained
*wR*(*F*^2^) = 0.242	*w* = 1/[σ^2^(*F*_o_^2^) + (0.1212*P*)^2^] where *P* = (*F*_o_^2^ + 2*F*_c_^2^)/3
*S* = 0.88	(Δ/σ)_max_ < 0.001
3708 reflections	Δρ_max_ = 0.25 e Å^−^^3^
263 parameters	Δρ_min_ = −0.22 e Å^−^^3^
0 restraints	Extinction correction: *SHELXL*, Fc^*^=kFc[1+0.001xFc^2^λ^3^/sin(2θ)]^-1/4^
Primary atom site location: structure-invariant direct methods	Extinction coefficient: 0.036 (5)

### Special details

**Table:**

Geometry. All e.s.d.'s (except the e.s.d. in the dihedral angle between two l.s. planes) are estimated using the full covariance matrix. The cell e.s.d.'s are taken into account individually in the estimation of e.s.d.'s in distances, angles and torsion angles; correlations between e.s.d.'s in cell parameters are only used when they are defined by crystal symmetry. An approximate (isotropic) treatment of cell e.s.d.'s is used for estimating e.s.d.'s involving l.s. planes.
Refinement. Refinement of *F*^2^ against ALL reflections. The weighted *R*-factor *wR* and goodness of fit *S* are based on *F*^2^, conventional *R*-factors *R* are based on *F*, with *F* set to zero for negative *F*^2^. The threshold expression of *F*^2^ > σ(*F*^2^) is used only for calculating *R*-factors(gt) *etc*. and is not relevant to the choice of reflections for refinement. *R*-factors based on *F*^2^ are statistically about twice as large as those based on *F*, and *R*- factors based on ALL data will be even larger.

### Fractional atomic coordinates and isotropic or equivalent isotropic displacement parameters (Å^2^)

**Table:**

	*x*	*y*	*z*	*U*_iso_*/*U*_eq_	
O1	0.2253 (10)	0.1891 (7)	0.26020 (13)	0.0951 (18)	
O2	−0.0875 (10)	0.1412 (7)	0.22882 (14)	0.0932 (17)	
O3	−0.1033 (8)	0.2666 (5)	0.10426 (11)	0.0669 (13)	
O4	0.1605 (8)	0.4177 (5)	0.07843 (12)	0.0703 (13)	
N1	0.1114 (12)	0.2008 (7)	0.23234 (16)	0.0725 (16)	
N2	0.0789 (10)	0.3493 (6)	0.10576 (14)	0.0558 (14)	
N3	0.5138 (9)	0.5396 (6)	0.11586 (14)	0.0559 (14)	
H03	0.4470	0.5385	0.0948	0.067*	
N4	0.7204 (9)	0.6275 (6)	0.12078 (13)	0.0561 (14)	
C1	0.4148 (10)	0.4550 (7)	0.14367 (16)	0.0475 (14)	
C2	0.2018 (10)	0.3647 (7)	0.13979 (15)	0.0481 (15)	
C3	0.1020 (12)	0.2848 (7)	0.16947 (17)	0.0554 (16)	
H3	−0.0398	0.2275	0.1671	0.066*	
C4	0.2148 (11)	0.2921 (8)	0.20181 (16)	0.0536 (16)	
C5	0.4246 (11)	0.3756 (8)	0.20701 (17)	0.0582 (17)	
H5	0.4983	0.3771	0.2296	0.070*	
C6	0.5215 (11)	0.4563 (8)	0.17795 (17)	0.0562 (16)	
H6	0.6626	0.5137	0.1811	0.067*	
C7	0.7784 (10)	0.7172 (7)	0.09321 (16)	0.0497 (15)	
H7	0.6795	0.7136	0.0730	0.060*	
C8	0.9857 (10)	0.8234 (7)	0.09117 (16)	0.0490 (15)	
C9	1.0328 (10)	0.8946 (7)	0.05641 (16)	0.0482 (15)	
C10	0.8823 (13)	0.8738 (8)	0.02552 (16)	0.0609 (17)	
H10	0.7477	0.8083	0.0274	0.073*	
C11	0.9330 (12)	0.9482 (8)	−0.00647 (18)	0.0667 (19)	
H11	0.8285	0.9355	−0.0258	0.080*	
C12	1.1380 (13)	1.0440 (8)	−0.0115 (2)	0.070 (2)	
H12	1.1724	1.0905	−0.0339	0.084*	
C13	1.2834 (13)	1.0664 (7)	0.01730 (19)	0.0652 (18)	
H13	1.4182	1.1309	0.0145	0.078*	
C14	1.2364 (12)	0.9944 (7)	0.05161 (17)	0.0519 (15)	
C15	1.3859 (11)	1.0226 (7)	0.08107 (18)	0.0583 (17)	
H15	1.5209	1.0862	0.0777	0.070*	
C21	1.1340 (10)	0.8580 (7)	0.12098 (15)	0.0466 (14)	
C16	1.3403 (10)	0.9587 (7)	0.11560 (17)	0.0525 (16)	
C17	1.4973 (12)	0.9911 (8)	0.14472 (18)	0.0591 (17)	
H17	1.6304	1.0560	0.1407	0.071*	
C18	1.4578 (12)	0.9301 (8)	0.1779 (2)	0.0660 (19)	
H18	1.5653	0.9491	0.1966	0.079*	
C19	1.2511 (12)	0.8366 (8)	0.18424 (18)	0.0635 (18)	
H19	1.2202	0.7970	0.2075	0.076*	
C20	1.0978 (12)	0.8040 (8)	0.15704 (17)	0.0604 (17)	
H20	0.9623	0.7433	0.1622	0.073*	

### Atomic displacement parameters (Å^2^)

**Table:**

	*U*^11^	*U*^22^	*U*^33^	*U*^12^	*U*^13^	*U*^23^
O1	0.097 (4)	0.130 (5)	0.059 (3)	−0.006 (4)	−0.007 (3)	0.025 (3)
O2	0.080 (4)	0.121 (4)	0.079 (3)	−0.027 (4)	0.010 (3)	0.016 (3)
O3	0.056 (3)	0.074 (3)	0.070 (3)	−0.017 (3)	−0.005 (2)	0.000 (2)
O4	0.069 (3)	0.085 (3)	0.057 (3)	−0.020 (3)	0.000 (2)	0.010 (2)
N1	0.071 (4)	0.086 (4)	0.061 (4)	0.004 (4)	0.007 (4)	0.004 (3)
N2	0.054 (3)	0.057 (3)	0.057 (3)	0.005 (3)	−0.004 (3)	−0.002 (3)
N3	0.053 (3)	0.055 (3)	0.059 (3)	−0.004 (3)	0.002 (3)	−0.001 (3)
N4	0.045 (3)	0.059 (3)	0.065 (3)	−0.001 (3)	0.002 (3)	0.001 (3)
C1	0.043 (3)	0.050 (3)	0.050 (4)	0.000 (3)	0.000 (3)	0.001 (3)
C2	0.044 (4)	0.052 (4)	0.048 (3)	−0.003 (3)	−0.002 (3)	0.002 (3)
C3	0.047 (3)	0.054 (4)	0.066 (4)	−0.002 (3)	0.008 (3)	−0.008 (3)
C4	0.052 (4)	0.065 (4)	0.045 (4)	−0.002 (4)	0.003 (3)	0.004 (3)
C5	0.056 (4)	0.068 (4)	0.051 (4)	0.006 (4)	−0.005 (3)	−0.004 (3)
C6	0.050 (4)	0.058 (4)	0.061 (4)	−0.004 (3)	−0.005 (3)	−0.001 (3)
C7	0.049 (4)	0.051 (4)	0.049 (3)	−0.001 (3)	0.003 (3)	−0.002 (3)
C8	0.044 (3)	0.039 (3)	0.064 (4)	−0.002 (3)	0.006 (3)	−0.001 (3)
C9	0.045 (3)	0.042 (3)	0.059 (4)	0.006 (3)	0.001 (3)	−0.001 (3)
C10	0.063 (4)	0.063 (4)	0.056 (4)	0.001 (4)	−0.002 (4)	0.002 (3)
C11	0.064 (5)	0.076 (5)	0.060 (4)	0.006 (4)	−0.004 (4)	0.005 (4)
C12	0.084 (5)	0.062 (4)	0.065 (4)	0.003 (4)	0.014 (4)	0.011 (4)
C13	0.072 (4)	0.045 (4)	0.079 (5)	−0.007 (4)	0.014 (4)	0.007 (3)
C14	0.058 (4)	0.040 (3)	0.058 (4)	−0.002 (3)	0.002 (3)	−0.001 (3)
C15	0.053 (4)	0.046 (4)	0.076 (5)	0.000 (3)	0.013 (4)	−0.002 (3)
C21	0.044 (3)	0.040 (3)	0.055 (4)	0.001 (3)	0.002 (3)	−0.003 (3)
C16	0.043 (4)	0.045 (3)	0.069 (4)	0.009 (3)	0.000 (3)	−0.007 (3)
C17	0.046 (4)	0.060 (4)	0.072 (5)	−0.002 (3)	−0.004 (4)	−0.006 (4)
C18	0.057 (4)	0.070 (5)	0.071 (5)	0.007 (4)	−0.010 (4)	−0.006 (4)
C19	0.067 (5)	0.065 (4)	0.059 (4)	0.007 (4)	−0.001 (4)	0.006 (3)
C20	0.057 (4)	0.058 (4)	0.067 (4)	−0.006 (4)	0.000 (4)	−0.001 (3)

### Geometric parameters (Å, º)

**Table:**

O1—N1	1.214 (7)	C9—C14	1.418 (8)
O2—N1	1.229 (7)	C9—C10	1.428 (8)
O3—N2	1.230 (6)	C10—C11	1.355 (8)
O4—N2	1.238 (6)	C10—H10	0.9300
N1—C4	1.469 (8)	C11—C12	1.407 (9)
N2—C2	1.436 (7)	C11—H11	0.9300
N3—C1	1.355 (7)	C12—C13	1.351 (9)
N3—N4	1.379 (6)	C12—H12	0.9300
N3—H03	0.8600	C13—C14	1.418 (8)
N4—C7	1.293 (7)	C13—H13	0.9300
C1—C6	1.397 (8)	C14—C15	1.392 (8)
C1—C2	1.416 (8)	C15—C16	1.397 (8)
C2—C3	1.390 (8)	C15—H15	0.9300
C3—C4	1.350 (8)	C21—C20	1.413 (7)
C3—H3	0.9300	C21—C16	1.437 (8)
C4—C5	1.378 (8)	C16—C17	1.414 (8)
C5—C6	1.369 (8)	C17—C18	1.337 (9)
C5—H5	0.9300	C17—H17	0.9300
C6—H6	0.9300	C18—C19	1.412 (9)
C7—C8	1.456 (7)	C18—H18	0.9300
C7—H7	0.9300	C19—C20	1.348 (8)
C8—C21	1.408 (8)	C19—H19	0.9300
C8—C9	1.430 (8)	C20—H20	0.9300
			
O1—N1—O2	122.7 (6)	C11—C10—C9	120.8 (7)
O1—N1—C4	118.4 (6)	C11—C10—H10	119.6
O2—N1—C4	118.8 (6)	C9—C10—H10	119.6
O3—N2—O4	121.4 (5)	C10—C11—C12	122.4 (7)
O3—N2—C2	119.3 (5)	C10—C11—H11	118.8
O4—N2—C2	119.3 (5)	C12—C11—H11	118.8
C1—N3—N4	120.9 (5)	C13—C12—C11	118.1 (6)
C1—N3—H03	119.6	C13—C12—H12	121.0
N4—N3—H03	119.6	C11—C12—H12	121.0
C7—N4—N3	113.9 (5)	C12—C13—C14	121.9 (7)
N3—C1—C6	120.0 (6)	C12—C13—H13	119.0
N3—C1—C2	122.6 (6)	C14—C13—H13	119.0
C6—C1—C2	117.4 (5)	C15—C14—C13	120.8 (6)
C3—C2—C1	120.5 (5)	C15—C14—C9	119.2 (6)
C3—C2—N2	116.7 (5)	C13—C14—C9	120.0 (6)
C1—C2—N2	122.8 (5)	C14—C15—C16	122.4 (6)
C4—C3—C2	118.8 (6)	C14—C15—H15	118.8
C4—C3—H3	120.6	C16—C15—H15	118.8
C2—C3—H3	120.6	C8—C21—C20	125.7 (6)
C3—C4—C5	123.2 (6)	C8—C21—C16	119.2 (5)
C3—C4—N1	117.7 (6)	C20—C21—C16	115.1 (6)
C5—C4—N1	119.0 (6)	C15—C16—C17	120.3 (6)
C6—C5—C4	118.1 (6)	C15—C16—C21	119.2 (6)
C6—C5—H5	120.9	C17—C16—C21	120.6 (6)
C4—C5—H5	120.9	C18—C17—C16	121.2 (6)
C5—C6—C1	122.0 (6)	C18—C17—H17	119.4
C5—C6—H6	119.0	C16—C17—H17	119.4
C1—C6—H6	119.0	C17—C18—C19	119.3 (6)
N4—C7—C8	125.5 (6)	C17—C18—H18	120.4
N4—C7—H7	117.3	C19—C18—H18	120.4
C8—C7—H7	117.3	C20—C19—C18	120.8 (6)
C21—C8—C9	120.4 (5)	C20—C19—H19	119.6
C21—C8—C7	123.7 (5)	C18—C19—H19	119.6
C9—C8—C7	115.9 (5)	C19—C20—C21	122.9 (6)
C14—C9—C10	116.7 (5)	C19—C20—H20	118.6
C14—C9—C8	119.7 (5)	C21—C20—H20	118.6
C10—C9—C8	123.6 (6)		

### Hydrogen-bond geometry (Å, º)

**Table:**

*D*—H···*A*	*D*—H	H···*A*	*D*···*A*	*D*—H···*A*
N3—H03···O4	0.86	1.99	2.617 (7)	129
C11—H11···O4^i^	0.93	2.47	3.251 (8)	142
C20—H20···N4	0.93	2.25	2.894 (8)	126

Symmetry code: (i) *x*+1/2, −*y*+3/2, −*z*.
